# Topical Application of Mesenchymal Stem Cell Exosomes Alleviates the Imiquimod Induced Psoriasis-Like Inflammation

**DOI:** 10.3390/ijms22020720

**Published:** 2021-01-13

**Authors:** Bin Zhang, Ruenn Chai Lai, Wei Kian Sim, Andre Boon Hwa Choo, Ellen Birgit Lane, Sai Kiang Lim

**Affiliations:** 1 Institute of Molecular and Cell Biology (IMCB)—A*STAR, 8A Biomedical Grove, #05-39 Immunos, Singapore 138648, Singapore; zhang_bin@imcb.a-star.edu.sg (B.Z.); Lai_Ruenn_Chai@imcb.a-star.edu.sg (R.C.L.); Eugene_Sim@imcb.a-star.edu.sg (W.K.S.); 2 Bioprocessing Technology Institute (BTI)—A*STAR, 20 Biopolis Way, Singapore 138668, Singapore; andre_choo@bti.a-star.edu.sg; 3 Skin Research Institute of Singapore (SRIS)—A*STAR, 8A Biomedical Grove, #06-06 Immunos, Singapore 138648, Singapore; birgit.lane@sris.a-star.edu.sg; 4 Department of Surgery, YLL School of Medicine, National University of Singapore c/o NUHS Tower Block, Level 8. IE Kent Ridge Road, Singapore 119228, Singapore

**Keywords:** mesenchymal stem cell, exosome, psoriasis

## Abstract

Severe psoriasis, a chronic inflammatory skin disease is increasingly being effectively managed by targeted immunotherapy but long-term immunotherapy poses health risk and loss of response. Therefore, there is a need for alternative therapy strategies. Mesenchymal stem/stromal cell (MSC) exosomes are widely known for their potent immunomodulatory properties. Here we investigated if topically applied MSC exosomes could alleviate psoriasis-associated inflammation. Topically applied fluorescent exosomes on human skin explants were confined primarily to the stratum corneum with <1% input fluorescence exiting the explant over a 24-h period. Nevertheless, topically applied MSC exosomes in a mouse model of imiquimod (IMQ) psoriasis significantly reduced IL-17 and terminal complement activation complex C5b-9 in the mouse skin. MSC exosomes were previously shown to inhibit complement activation, specifically C5b-9 complex formation through CD59. Infiltration of neutrophils into the stratum corneum is characteristic of psoriasis and neutrophils are a major cellular source of IL-17 in psoriasis through the release of neutrophil extracellular traps (NETs). We propose that topically applied MSC exosomes inhibit complement activation in the stratum corneum and this alleviates IL-17 release by NETS from neutrophils that accumulate in and beneath the stratum corneum.

## 1. Introduction

Psoriasis is a common chronic disease involving predominantly the skin and joints, and is associated with underlying genetic predispositions and inflammatory dysregulation [1]. Although there is no cure for psoriasis, there are well-established management protocols. The first line therapy for mild and moderate psoriasis is usually topical therapy that involves a combination of glucocorticoids, vitamin D analogues, and phototherapy, while that for moderate to severe psoriasis often requires systemic therapy [2,3,4]. Drugs used in systemic therapy for psoriasis include both small molecules such as methotrexate (MTX), retinoids, or cyclosporin, and more recently, biologics such as monoclonal antibodies and receptor fusion proteins. Most of the small molecules reduce cellular proliferation or suppress immune activity while the biologics target inflammatory cytokines in the IL-23/Th17 axis and TNF-α-signaling [4]. However, long-term use of these systemic treatments is often complicated by toxic side effects such as hepatotoxicity, nephrotoxicity, hypertension, tremors, hypomagnesemia, hyperkalemia, and malignancies [5,6,7], inadequate long term patient compliance [8] and anti-drug responses [9,10]. Therefore, there is still a need for alternative treatment modalities for at-risk or non-responsive patients.

Exosomes derived from mesenchymal stem cells (MSCs) are a potential treatment alternative. MSCs are the most extensively clinically trialed cells with an established record of safety in human patients and are currently approved for use in highly intractable inflammatory disorders such as acute graft versus host disease (GVHD) and Crohn’s related enterocutaneous fistula disease [11]. Although the therapeutic efficacy of MSCs was initially predicated on the migration of transplanted MSCs to injured or diseased tissues where they engraft and differentiate to form new replacement tissues, small numbers of transplanted MSCs were observed to engraft or differentiate in biologically relevant numbers in affected tissues even when there were functional improvements. Based on the prolific secretion of small bioactive molecules by MSCs [12], Caplan and Dennis proposed in 2006 that MSCs mediate their therapeutic activity via secretion rather than by direct cellular interactions [13]. In 2007, it was first reported that the active agent in cardioprotective MSC secretion was not a small molecule but a large complex with a MW larger than 1000 kDa [14]; and this agent was subsequently found to be lipid membrane vesicles i.e., exosomes of 110–130 nm in diameter [15]. Independently, Bruno et al. also reported that MSC microvesicles, which are secreted membrane vesicles of 80 nm to 1 μm, improve acute kidney injury in a mouse model [16]. Today, it is widely accepted that MSC exosomes or small extracellular vesicles (sEVs) of 50–200 nm mediate the therapeutic potency of MSCs [17]. 

Exosomes are traditionally defined as vesicles derived from endosomal multivesicular bodies. In this study, we used sEV preparations that were previously shown to contain exosomes i.e., small EVs with an endosomal biogenesis and carried exosome-associated markers [18]. These exosomes in this study were prepared from a clonal immortalized MSC cell line and we have shown that the exosomes produced by MSC before and after immortalization were highly similar [19]. We have previously characterized these exosomes as having a hydrodynamic radius of 55–65 nm with lipid membranes composed of cholesterol, sphingomyelin, and phosphatidylcholine using standard biochemical assays [15,20]. These exosomes were determined to have a protein-rich cargo of ~1000 proteins that include exosome-associated proteins, e.g., CD81, CD9, and Alix through western blot analysis, and a combination of mass spectrometry and antibody array [15,20,21]. They also have a diverse RNA cargo as determined by array hybridisation, RT-PCR and RNA sequencing [22,23]. Transmission electron microscopy (TEM) and immunoelectron microscopy analysis confirmed the presence of vesicular particles of ~100–200 nm [23] and the presence of CD81 on the surface membrane of these vesicles [24], respectively. These MSC exosome preparations are highly immunomodulatory and can attenuate inflammation by enhancing secretion of anti-inflammatory cytokines, promoting Treg polarization and inhibiting complement activation [20,25,26]. They have also been shown to inhibit complement activation and the formation of C5b-9 complex, the terminal complement complex (TCC) through CD59 present in MSC exosomes [20].

We investigated the possibility of applying MSC exosomes as a topical application to alleviate local skin inflammation seen in psoriasis. Psoriasis is an inflammatory disease that manifests as localized skin lesions, and identification of an effective topical therapeutic may reduce the risks of systemic adverse side effects and possible anti-drug responses. Topical application of drugs could potentially bypass several issues associated with oral or intravenous administration such as first-pass metabolism, resulting in higher bioavailability to the target area allowing reduced dosing quantum frequency, leading to reduced side effects and improved patient compliance [27].

Inflammation in psoriasis involves complex interplay between the innate and adaptive immune systems. Although the way these two systems interact is unclear, the cytokine members in the IL-23/IL-17 axis have been shown to be critical to the pathogenesis of psoriasis [28,29]. In addition, complement components which constitute a major first line of immune defense and mediate the interaction of the innate and adaptive immune systems have also long been implicated in psoriatic inflammation as reviewed [30]. Based on these observations, we investigated the effect of topically applied MSC exosomes on IL-17, IL-23 and C5b-9 in the skin of a mouse model of psoriasis induced by imiquimod (IMQ). 

## 2. Results

### 2.1. Effect of Topically Applied MSC Exosomes on the Skin Phenotype of A Mouse Model of IMQ-Induced Psoriasis Inflammation

**Figure 1:**
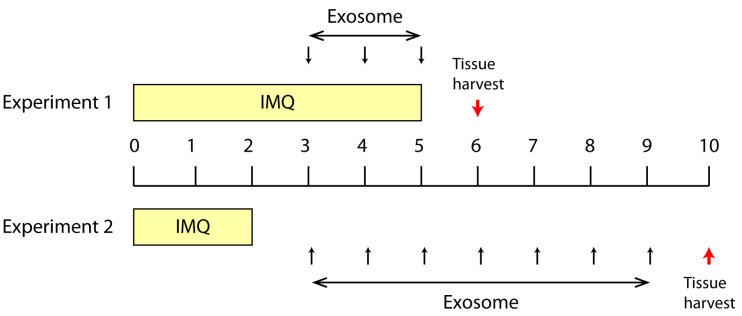
Summary of two treatment protocols in the Imiquimod (IMQ) induced psoriasis mouse model. In Expt.1, IMQ was topically applied from Day 0 to Day 5 and exosome cream was applied from Day 3 to Day 5. The experiment was terminated on day 6. In Expt. 2, IMQ was topically applied from Day 0 to Day 2 and exosome cream was applied from Day 3 to Day 9. The experiment was terminated on Day 10. The red arrow indicated the study termination day. This experiment was performed twice with two independently prepared batches of exosomes.

**Figure 2:**
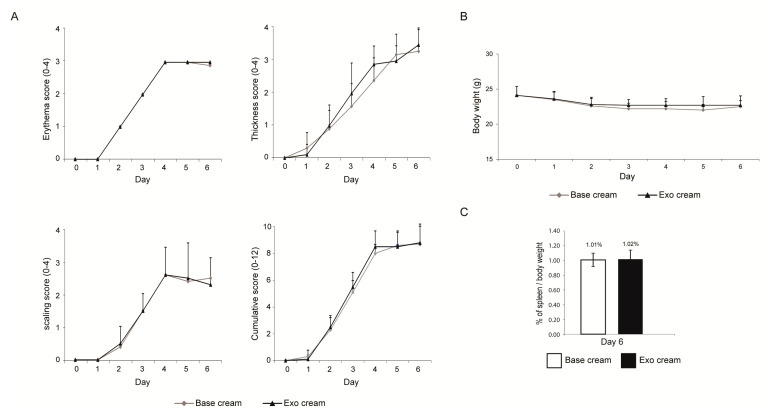
Skin phenotype score, body weight and ratio of spleen to body weight. (**A**) Erythema, scaling, and thickness of the back skin were scored independently daily from Day 0 to Day 6 (Expt. 1) on a scale from 0 to 4, and the cumulative score for erythema, scaling, and thickness over time was determined daily on a scale from 0–12. (**B**) The body weight (Day 0–6) and (**C**) ratio of spleen to body weight (Day 6) were also determined. Scoring (erythema, scaling, thickness, and cumulative score), body weights and ratio of spleen to body weight were analyzed using Student’s *t*-test. *p* values < 0.05 were considered as statistically significant.

To test the effect of topically applied MSC exosomes on psoriatic skin inflammation, we first applied IMQ to mouse skin for 6 days (Day 0 to Day 5) to induce psoriatic skin lesions, and then applied the MSC exosome preparation for three days on Day 3 to Day 5 (Experiment 1, Figure 1). The rationale for not initiating topical application of MSC exosomes from the first day of IMQ induction, as is commonly practiced, was to allow assessment of the effect of MSC exosomes on the psoriatic phenotype while minimizing any confounding effects on the induction of psoriasis by IMQ. The skin phenotype as measured by erythema, scaling and thickness were similarly severe in both control (base cream) and treatment (exosome cream) groups as indicated by a cumulative score of 12 on Day 0 to Day 6 (Figure 2A). Though the body weight was normal (Figure 2B), the spleen/body weight ratio was elevated at 1% against the average of 0.28% for male Balb/C mice [31] (Figure 2C). Using this protocol however, exosome treatment did not elicit a significant difference in the level of IL-17, IL-23 and C5b-9 (Expt1, Figure 4). This may have been due to the severe disease phenotype and/or short treatment duration.

**Figure 3:**
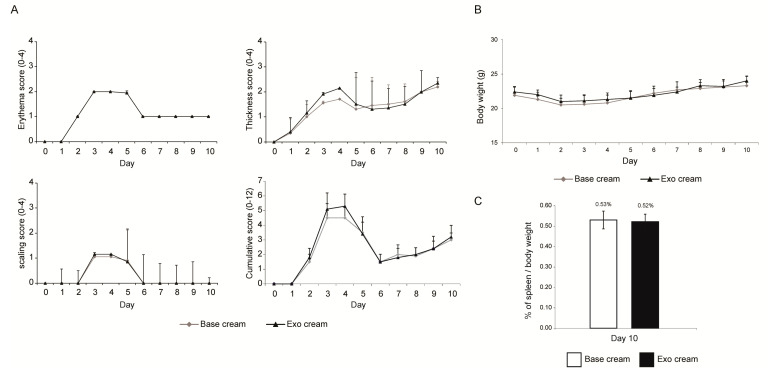
Skin phenotype score, body weight and ratio of spleen to body weight. (**A**) Erythema, scaling, and thickness of the back skin were scored independently daily from Day 0 to Day10 (Expt. 2) on a scale from 0 to 4, and the cumulative score for erythema, scaling, and thickness over time was determined daily on a scale from 0–12. (**B**) The body weight (Day 0–10) and (**C**) ratio of spleen to body weight (Day 10) were also determined. Two independent animal experiments using different batches of exosome preparations were performed. The scores from the two independent studies were combined. Scoring (erythema, scaling, thickness, and cumulative score), body weights and ratio of spleen to body weight were analyzed using Student’s *t*-test. *p* values < 0.05 were considered as statistically significant.

**Figure 4:**
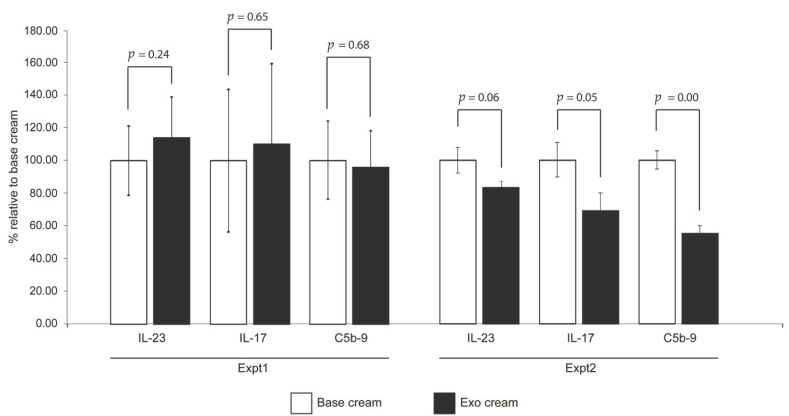
Cytokine induction and complement activation. The shaved back skin of each mouse was removed on day 6 (Expt. 1) or day 10 (Expt. 2), and assayed for IL-17, IL-23 and C5b-9 terminal complement complex (TCC). The levels of cytokines and TCC in “exo cream” (with MSC exsomes) was normalized to that of the “base cream” (vehicle control). Expt. 1 was performed with one independent animal experiment, and Expt. 2 was performed with two independent studies and combined. Statistical significance was determined by Student’s *t* test. *p* values < 0.05 were considered as statistically significant.

In the second experiment, IMQ was applied for 3 days only (Day 0, 1 and 2) to induce milder psoriatic skin lesions before applying MSC exosomes for seven days from Day 3 to Day 9 (Experiment 2, Figure 1). Consistent with the reduced IMQ application, the psoriatic phenotype was less severe at ~50% maximal score on day 3, a day after IMQ application was stopped (Figure 3A). With the relatively mild skin phenotype, the control (base cream) mice returned nearly to the baseline by the end of the experiment, thus obviating a need for therapeutic intervention to improve skin phenotype. As expected, exosome treatment did not improve the recovery (Figure 3A). The body weight was similar in both treated and untreated animals and within the normal range [31] (Figure 3B), but the spleen/body weight ratio was elevated by 0.53% in untreated mice and 0.52% in exosome-treated mice against the average of 0.28% for male Balb/C mice [31] (Figure 3C). This suggests that despite the mild skin phenotype, both control and treated animals showed an activated immune system and were in an inflammatory state. The spleen/body weight ratio in exosome-treated animals was reduced compared to the untreated mice but the reduction was not statistically significant. Nevertheless, IL-17, IL-23 and C5b-9 complex were reduced in the skin of mice treated with the exosome cream relative to those treated with base cream (Expt.2, Figure 4), and the reductions for IL-17 and C5b-9 complex were statistically significant at *p* ≤ 0.05.

### 2.2. Dermal Penetrance of Topically Applied Exosomes on Human Skin

**Figure 5:**
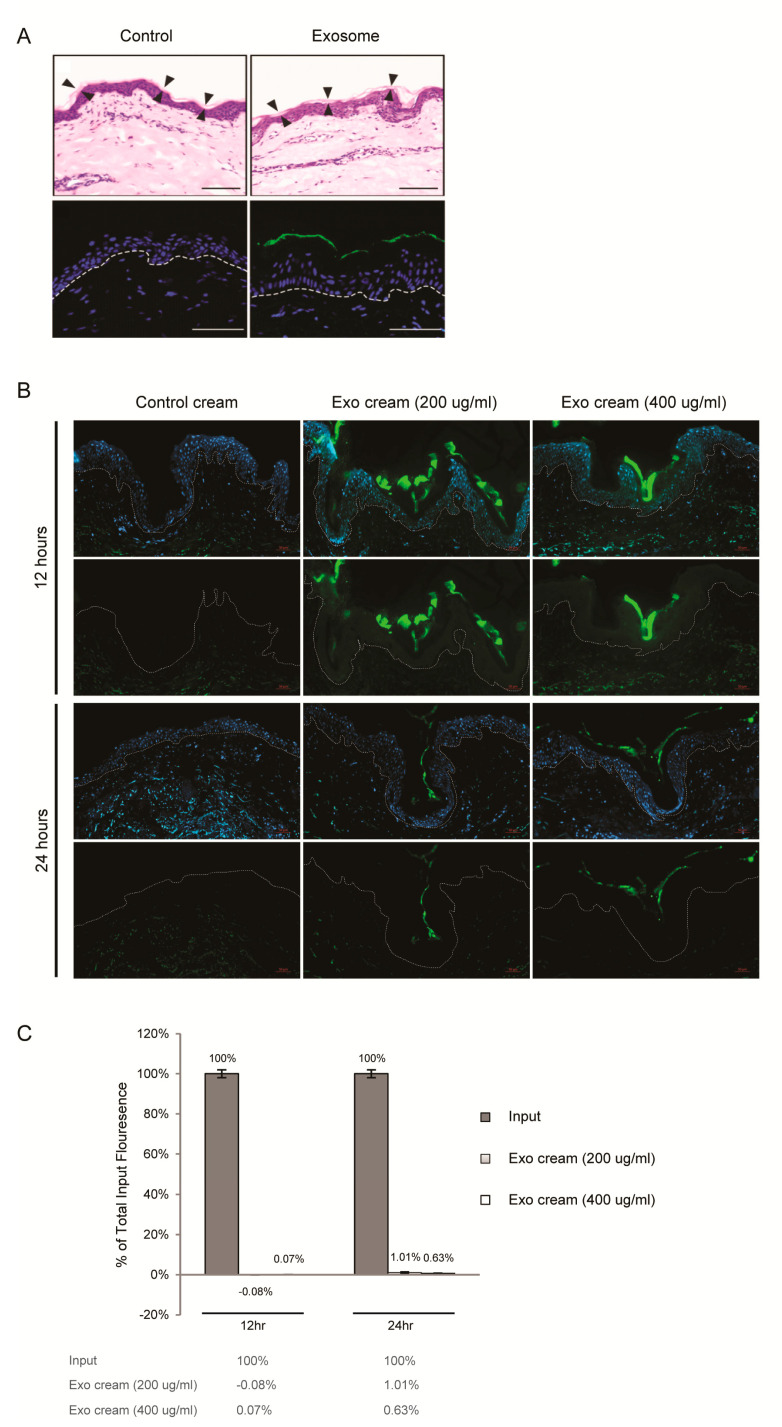
Dermal penetrance of MSC exosomes (**A**) Topical application of MSC exosomes (aqueous) on human skin explant culture. Human skin explant was topically treated with PBS (Control) or Alexa Fluor 488-labeled exosomes, washed, frozen in OCT medium and sectioned. (Upper panel) H&E staining with arrowheads indicating boundary of the stratum corneum. Scale bar = 100 μm. (Lower panel) Fluorescence imaging of sections counterstained with DAPI (blue) to show nuclei. White broken lines denote epidermal-dermal junction. Scale bar = 50 μm. (**B**) Topical application of exosome oil-in-water emulsion cream on skin explant culture. Human skin explant culture was topically treated with AF488-labeled PBS (control cream) or AF488-labeled exosome cream (200 μg/mL or 400 μg/mL) for 12 and 24 h, washed, frozen in OCT medium and sectioned. Fluorescence imaging of sections counterstained with DAPI (blue, upper panel) or without DAPI (lower panel). White broken lines denote epidermal-dermal junction. Scale bar = 50 μm (**C**) Relative AF488 signal in culture medium of skin explants to input fluorescence. After subtracting the AF488 signal in the label control cream, the fluorescence signal in the culture of explants treated with exosome cream (200 μg/mL or 400 μg/mL) was normalized to their respective input signals to calculate the percentage of total input fluorescence in culture medium of skin explants.

Investigations have demonstrated that skin penetration by nanoparticles such as liposomes is highly inefficient [32]. In fact, liposomes have been observed to disintegrate in the stratum corneum [33]. To assess the dermal penetrance of MSC exosomes, MSC exosomes were covalently labeled with a fluorescent dye and applied topically to the surface of intact human skin in explant cultures. Within 2 h, fluorescence was seen to localize mainly to the stratum corneum and we did not observe any fluorescence in the underlying nucleated stratum granulosum (Figure 5A). To assess the persistence and penetrance of the exosomes in human skin, explants and culture media were harvested at 0, 12 and 24 h. In cryosections, fluorescence was again largely confined to the stratum corneum and was maximum at 12 h (Figure 5B). Again, little or no fluorescence was observed in the underlying nucleated stratum granulosum at 12 or 24 h. Consistent with this observation, total fluorescence in the culture medium harvested at 12 and 24 h was negligible and <1% of input, respectively (Figure 5C).

## 3. Discussion

In this study, we demonstrated that topically applied MSC exosomes can reduce the critical psoriatic cytokines, IL-17 and IL-23, and terminal complement complex, C5b-9. This is detectable even in a mild psoriatic phenotype induced by 3 days IMQ treatment of mouse skin. IL-17 and IL-23 [28,34,35] are the major cytokines implicated in the pathogenesis of psoriasis and are also the major therapeutic targets in the treatment of psoriasis [8]. The importance of these cytokines in mediating the inflammatory phenotype is reflected in their involvement in other inflammatory skin disorders including atopic dermatitis [36], diabetic wound [37,38], contact dermatitis [39,40,41], acne [42], and keloids [43]. However, we have also shown here that when topically applied, either in an aqueous solution or in oil-in-water emulsion cream, MSC exosomes remain largely confined to the stratum corneum of human skin explant, as would be predicted from their liposome-like nature [23]. They are prevented from further ingression into the epidermis by the layers of tight junctions found in the underlying stratum granulosum [44]. The milieu of the stratum corneum is rich in enzymes such as proteases [45,46], phospholipases [47], RNases [48] and DNases [49], and the fluorescence detected in the culture medium may reflect degraded exosomes. Nevertheless, there are many reports of liposomal carriers enhancing the efficacy of drugs for management of skin diseases [50], suggesting that entry only into the stratum corneum may provide sufficient access to enable a number of potent biological effects. 

Since topically applied exosomes cannot overcome the skin barrier to access the living cells in the epidermis, their primary site of action must lie within the stratum corneum. A likely target of MSC exosomes is the complement system. Complement components are known to be present in the stratum corneum [30], and complement activation has been associated with psoriasis since the 1970s [30], although recent studies of the immunopathology of psoriasis have focused largely on Th17 cytokines and T cells [51]. In the early 1970s, highly chemotactic activated complement components C3a and C5a were found to be elevated in psoriatic plaque relative to non-psoriatic scale [52,53,54,55]. The terminal complement complex C5b-9 was also found to be present in psoriatic lesional plaques but not in non-psoriatic plaque tissues [56]. The chemotactic activated complement components have been shown to be responsible for neutrophil migration into the stratum corneum to form Munro microabscesses characteristic of psoriasis [52,53,54,55]. Notably, Munro microabscesses are also characteristic of IMQ-induced psoriasis in the mouse model where neutrophils were observed to accumulate just beneath the stratum corneum with some infiltration into the stratum corneum [57]. The signals for neutrophil migration, initiated within the sealed-off stratum corneum compartment, are then transmitted to the immune system e.g., by the Langerhans cells. These cells are epidermal dendritic cells of the immune system, whose dendrites when activated can extend up through the stratum granulosum tight junctions to sample the antigenic milieu of the stratum corneum without breaking the seal of the skin barrier [58].

Neutrophils are now considered a major cell source of IL-17 in psoriasis, through the release of neutrophil extracellular traps (NETs) during NETosis [59]. NETosis is increased in psoriasis [60], and has been shown to induce neutrophils and mast cells to secrete IL-17 [61]. One of the major inducers of NETosis is activated complement [62]. We have previously shown that MSC exosomes can inhibit complement activation through the inhibition of C5b-9 complex formation and this inhibition was abrogated by a neutralizing antibody against CD59 [20]. 

**Figure 6:**
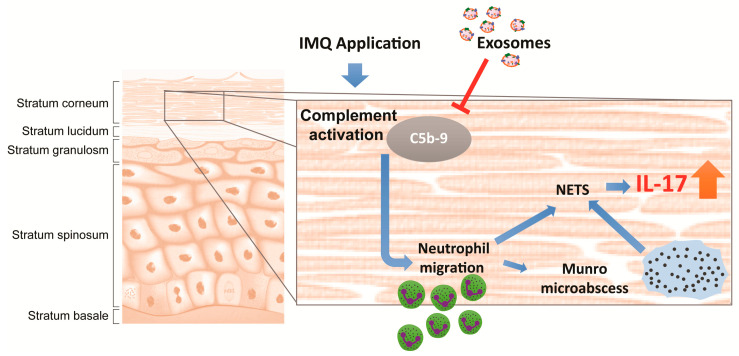
Illustration showing topically applied MSC exosomes localize to the stratum corneum, where they inhibit complement activation caused by the IMQ application, leading to attenuation of NETosis and subsequent release of IL-17 through neutrophil extracellular traps (NET). The T arrow indicated the inhibition of C5b-9 complex assembly by exosomes.

Based on these observations, we propose that topically applied MSC exosomes will permeate the depth of the stratum corneum where they inhibit complement activation through CD59 present on the exosomes [20], leading to attenuation of NETosis in neutrophils accumulated in but mostly just beneath the stratum corneum and thus reducing the release of IL-17 through NETs (Figure 6). Consistent with this hypothesis, topical application of MSC exosomes in a mouse model of IMQ-induced psoriasis resulted in the reduction of C5b-9 and IL-17. The stratum corneum is in fact a highly immune reactive zone [63], and the localization of topically applied MSC exosomes to the stratum corneum may effectively alleviate immune reactivity in the stratum corneum. As activation of complements and neutrophils represents the first line of immune defence, their attenuation could also alleviate the propagation and amplification of the inflammatory signals throughout the psoriatic skin.

## 4. Materials and Methods 

### 4.1. Preparation of MSC Exosomes

Immortalized E1-MYC 16.3 human ESC-derived mesenchymal stem cells were cultured in DMEM with 10% fetal calf serum as previously described [19]. For MSC-sEV preparation, the conditioned medium was prepared by growing 80% confluent cells in a chemically defined medium for three days as previously described [15,64,65]. The defined medium was prepared as follows: 480 mL DMEM (31053, Thermo Fisher, Waltham, MA, USA), 5 mL NEAA (11140-050, Thermo Fisher, Waltham, MA, USA), 5 mL L Glutamine (25030-081, Thermo Fisher, Waltham, MA, USA), 5 mL Sodium Pyruvate (11360, Thermo Fisher, Waltham, MA, USA), 5 mL ITS-X (51500-056, Thermo Fisher, Waltham, MA, USA), 0.5 mL 2-ME (21985-02, Thermo Fisher, Waltham, MA, USA). This was supplemented with 0.1 mL bFGF (0.5 ng/μL 0.2% BSA in PBS (+) and 0.005 mL PDGF (100 ng/μL PBS (+)). These latter components were obtained as follows: Bovine Serum Albumin or BSA (A9647, Sigma-Aldrich, St. Louis, MO, USA), PDGF (100-00 AB Cytolab Ltd., Rehovot, Israel), bFGF (13256-029, Thermo Fisher, Waltham, MA, USA) and PBS (+) (14040-133, Thermo Fisher, Waltham, MA, USA). The conditioned medium (CM) was size-fractionated by tangential flow filtration and then concentrated 50× using a membrane with a molecular weight cut-off (MWCO) of 100 kDa (Sartorius, Gottingen, Germany). The MSC-sEV preparation was assayed for protein concentration using a Coomassie Plus (Bradford) Assay Kit (ThermoFisher, Waltham, MA, USA). Only batches of sEV determined by Nanoparticle tracking analysis on a ZetaView instrument (Particle Matrix GmbH, Inning am Ammersee, Bavaria, Germany) to have 1.46 × 10^11^ ± 2.43 × 10^10^ particles per ug protein and particle modal size of 138.62 ± 4.45 nm using the parameters (sensitivity = 90, shutter = 70, frame rate = 30, min brightness = 25, min area = 5, max area = 1000) were used for this study. In addition, preparations must express CD81 and CD73 as determined by western or ELISA [15,18,19,20,21,22,23,24]. The sEV preparations were filtered with a 0.22μm filter (Merck Millipore, Billerica, MA, USA) and stored in −20 °C freezer.

### 4.2. Formulation of Oil-in-Water Emulsion of MSC Exosomes 

The oil-in-water emulsion of MSC exosome was composed of ingredients in Table 1. For skin penetrance studies, the exosomes were fluorescence-labeled with Alexa Fluor 488 as described below. Just before use, the mixture was emulsified by placing the mixture in a two-syringe set up connected by a tubing and mixed by alternately pushing the syringe plungers 20 times so that content in one syringe was pushed into the other in an alternate fashion.

### 4.3. Fluorescence Labeling of MSC Exosomes 

MSC exosomes were labeled with Alexa Fluor 488 amine-reactive probe (#A30005, ThermoFisher, Waltham, MA, USA) according to the manufacturer’s protocol. 1 mg of MSC exosomes in 0.8 mL PBS or 0.8 mL PBS were incubated with 1 mg of the Alexa Fluor 488 probe in a final volume of 1 mL 0.1 M sodium bicarbonate buffer for 1 h with gentle shaking and protected from light. Excess unreacted probes were removed by passing the two mixtures through Bio-Gel P30 gel columns (#7326231, Bio-Rad Laboratories, Hercules, CA, USA). The respective filtrates representing the labeled exosomes and the label control were sterile filtered through 0.22 μm filters. The labelled exosomes or label control were used in an oil in water emulsion as described below. The final exosome concentration the creams used for penetrance was 0 µg exosomes (Label Control Cream), 200 µg exosomes and 400 µg exosomes per ml cream. 

### 4.4. IMQ Induced Psoriasis Mouse Model

This study was performed by Washington Biotechnology INC, 6200 Seaforth Street Baltimore, MD 21224 under IACUC no: 17-003. On day 0, 20 mice (6–9 weeks, Balb/c, male, 10 mice per group) were tagged (left ear) for individual identification, weighed, and 1.5 cm × 2 cm of the back skin was shaved for IMQ induction. All mice were kept in quarantine for 3 weeks before starting the experiment. The exosome cream was prepared as above and the control base cream was the exosome cream without the exosomes. A psoriatic phenotype was induced by daily topical applications of 50 mg IMQ cream (5% Aldara cream) on the shaved back on day 0–5 or day 0–2 (total 6 or 3 doses, labeled as experiment 1 or 2). Daily topical application of base and exosome was initiated on day 3 and continued for a total of three or seven days (dosage: 100 µg/mL, 200 µL per mouse, total 3 or 7 doses, labeled as experiment 1 or 2) (Figure 1). Erythema, scaling, and thickness of the back skin were scored independently daily on a scale from 0 to 4 [57]. The back skin thickness was measured by electronic calipers as an indicator of edema. The shaved back skin was collected from each animal at termination (day 6 or 10), weighed and homogenized for cytokines (IL-17 and IL-23) and TCC (C5b-9) measurement. Scoring (erythema, scaling, thickness, and cumulative score), thickness measurements, spleen weights and cytokine results were analyzed using Student’s *t*-test (Microsoft Excel 2013, two-tailed). *p* values < 0.05 were considered as statistically significant. In the control arm, mice were treated with just the base cream (vehicle control). Non-IMQ induced skin adjacent to the IMQ induced skin in the control animals were removed to determine baseline cytokine levels in non-IMQ induced skin.

### 4.5. IL-17, IL-23 and C5b-9 Measurement Assays 

This study was performed by Washington Biotechnology INC, 6200 Seaforth Street Baltimore, MD 21224. The skin samples were thawed and cut to pieces in the 15 mL-tube before homogenizing in 2 mL of tris buffer plus 10 µL protease inhibitor (#P8340, Sigma-Aldrich, St. Louis, MO, USA) in 4 °C for 2 min. After homogenizing, the samples were centrifuged (14,000 rpm, 5 min, 4 °C), and the supernatants were collected for measurement of cytokines (IL-17, IL-23) and TCC (C5b-9) using commercially available ELISA kits (#M1700 and M2300 respectively, R&D Systems, Minneapolis, MN, USA; #MBS2021342, MyBioSource, San Diego, CA, USA) according to the manufacturer’s protocol. The results were analyzed using Student’s *t*-test. *p* values < 0.05 was considered as statistically significance.

### 4.6. Human Skin Penetrance Assay

The labeled MSC exosomes or Label control oil-in-water emulsion creams were applied to human skin explant to assess the penetrance of topically applied exosomes. The final exosome concentrations used in this study were 0 (label control), 200 and 400 µg exosomes per ml cream. The penetrance study was performed by DeNova Sciences Pte Ltd. Full thickness discarded human skin from abdominoplasty procedure was obtained with informed patient consent and proper DSRB approval (DeNova Sciences; NHG DSRB 2016/00525, 8 September 2016). The hypodermis/fats were removed and only the intact skin consisting of dermis and epidermis was kept. After that, the skin was soaked in 70% ethanol for 2 s to remove contamination, rinsed in 1× PBS for 3 min, and then placed in DeNova decontamination medium for 2 hrs in 37 °C CO_2_ incubator. Thereafter, the skin explants were cut into 0.5 by 2.5 cm strips. The strips have an average thickness of 0.45 ± 0.05 cm and surface area of 1.0 cm^2^. The strips were placed with the apical epithelial surface up in the Transwell permeable supports of a 6 well plate with 1 mL of DeNova skin explant medium maintained at the air-medium interface. 20 μL of PBS and 20 μL of label control, or 200 or 400 µg/mL labeled MSC exosome cream were each then applied to the apical surface of the strips using a positive dispenser pipette. The strips were incubated for 0, 12 or 24 h in a 37 °C incubator. At the end of each incubation period, the culture media were collected and the skins were gently washed with PBS. The strips were blotted dry and then placed in OCT quick-freeze medium compound, snap-frozen in liquid nitrogen and sectioned. 3 μm sections were mounted on glass slides and subjected to hematoxylin and eosin staining (H&E) analysis and fluorescence screening. Images were taken using EVOS microscope for H&E and Carl Zeiss microscope for fluorescence (DAPI and AF488 detection). The fluorescence in the culture media was measured in a fluorescence microplate reader (BioTek Synergy H1, BioTek, Winooski, VT, USA). 100 µL of culture medium representing 10% of each differently treated skin explant culture medium was used for the measurement. AF488 labeled-exosomes equivalent to 10% of the input was used as the respective input reference. The signal was then read at EX/EM 480 nm/520 nm. The AF488 labeled-exosomes were used to generate a standard curve to estimate the amount of AF488 signal in the skin explant culture medium.

## Figures and Tables

**Table 1:** Formulation of oil-in-water emulsion of MSC exosomes.

	Volume or Weight	Final Concentration
Olive oil	200 μL	20% *v*/*v*
Exosomes	200 or 400 μg	200 or 400 μg/ml
100% Seppic plus 400 ^1^	40 mg	4% *w*/*v*
PBS	400 μL	40% *v*/*v*
water	400 μL	40% *v*/*v*

^1^ Seppic 400 is a commercial emulsifying agent consisting of Polyacrylate-13, Polyisobutene & Polysorbate 20 sold by Seppic, https://www.seppic.com.

## Data Availability

Data is contained within the article.

## Abbreviations

MSC	Mesenchymal stem/stromal cell
IMQ	Imiquimod
TCC	Terminal complement complex
NET	Neutrophil extracellular traps
